# Preparation of a new adsorbent from activated carbon and carbon nanofiber (AC/CNF) for manufacturing organic-vacbpour respirator cartridge

**DOI:** 10.1186/1735-2746-10-15

**Published:** 2013-01-26

**Authors:** Mehdi Jahangiri, Javad Adl, Seyyed Jamaleddin Shahtaheri, Alimorad Rashidi, Amir Ghorbanali, Hossein Kakooe, Abbas Rahimi Forushani, Mohammad Reza Ganjali

**Affiliations:** 1 Department of Occupational Health, School of Public Health and Nutrition, Shiraz University of Medical Sciences, Shiraz, Iran; 2 Department of Occupational Health, School of Public Health, Tehran University of Medical Sciences, Tehran, Iran; 3 Departments of Occupational Health, School of Public Health, and Institute for Environmental Research, Tehran University of Medical Sciences, Tehran, Iran; 4 Research Institute of Petroleum Industry (RIPI), Tehran, Iran; 5 Department of Chemical Engineering, Faculty of Engineering, University of Tehran, Tehran, Iran; 6 Department of Biostatistics, School of Public Health, Tehran University of Medical Sciences, Tehran, Iran; 7 Center of Excellence in Electrochemistry, Faculty of Chemistry, University of Tehran, Tehran, Iran

**Keywords:** Carbon nanofibers, Respirator, Cartridge, Activated carbon, Organic-vapor

## Abstract

In this study a composite of activated carbon and carbon nanofiber (AC/CNF) was prepared to improve the performance of activated carbon (AC) for adsorption of volatile organic compounds (VOCs) and its utilization for respirator cartridges. Activated carbon was impregnated with a nickel nitrate catalyst precursor and carbon nanofibers (CNF) were deposited directly on the AC surface using catalytic chemical vapor deposition. Deposited CNFs on catalyst particles in AC micropores, were activated by CO_2_ to recover the surface area and micropores. Surface and textural characterizations of the prepared composites were investigated using Brunauer, Emmett and Teller’s (BET) technique and electron microscopy respectively. Prepared composite adsorbent was tested for benzene, toluene and xylene (BTX) adsorption and then employed in an organic respirator cartridge in granular form. Adsorption studies were conducted by passing air samples through the adsorbents in a glass column at an adjustable flow rate. Finally, any adsorbed species not retained by the adsorbents in the column were trapped in a charcoal sorbent tube and analyzed by gas chromatography. CNFs with a very thin diameter of about 10-20 nm were formed uniformly on the AC/CNF. The breakthrough time for cartridges prepared with CO_2_ activated AC/CNF was 117 minutes which are significantly longer than for those cartridges prepared with walnut shell- based activated carbon with the same weight of adsorbents. This study showed that a granular form CO_2_ activated AC/CNF composite could be a very effective alternate adsorbent for respirator cartridges due to its larger adsorption capacities and lower weight.

## Introduction

Volatile organic compounds (VOCs) are a class of chemical pollutants in the environment that can cause severe health problems. Some VOCs, like benzene, toluene and xylene (BTX) are known or suspected as carcinogens [1,2].

Adsorption of contaminants on sorbents is one of the most important methods for controlling VOC emissions. Activated carbon (AC) is the most versatile and frequently used sorbent for environmental control in the form of a fixed bed. This is due to its large internal surface area and pore volume, and its ability to absorb organic vapors at low cost. These characteristics make it one of the most practical adsorbents [3,4] for removing toxic gases and vapors from inhaled air via air-purifying respirators or personal protection devices. Respirator cartridges and canisters contain activated carbon granules in a packed bed which adsorb organic vapors from air while passed through the sorbents [5]. The typical organic vapor half-mask respirator cartridge is a plastic or metal case containing 25-40 g of activated carbon which is sometimes impregnated with metallic salt to enhance its acid gas adsorption characteristics.

Activated carbon is structureless and has complex physicochemical properties [6]. It has poor selectivity, especially for aromatic compounds [7]. The adsorptive capacity of activated carbon is limited by the following factors: the density of surface active sites, the activation energy of adsorptive bonds, the slow kinetics and non-equilibrium of sorption in heterogeneous systems, the mass transfer rate to the sorbent surface and its relatively large dimensions [8].

To overcome the above restrictions, new classes of synthesized carbonaceous materials have been proposed as candidates, or as modifying factors, for improving the adsorption properties of activated carbon.

In recent years, carbon nanofibers (CNFs) have received growing attention and been extensively studied. Because of their interesting properties (purity, mechanical strength, high geometrical surface, etc.) [9-12] they are recognised as a unique carbon material and as having potential for use as adsorbents and catalyst supports.

CNFs are characterized by a graphite-like structure at the nano-scale, where variable alignments of laminated hexagonal layers along the fibre’s axis typically provide three types of CNF: platelet (alignment perpendicularly to the fibre’s axis), tubular (alignment parallel to the axis), and herringbone (alignment angled to the axis) CNF[11]. These structures differ according to their growing conditions and the metal used as a catalyst [13].

Several studies have been conducted using CNF to improve the properties of other adsorbents. For example, CNF/carbon fibre composites have been investigated to improve adhesive properties at the interface between the fibres and the matrix in carbon fibre reinforced composites [14-16]. They have also been used to give a macroscopic frame or support to be anchored by CNFs to solve handling difficulty and the pressure drop problem [17]. Lim *et al.,*[12] used CNF/ACF (activated carbon fibre) composites to introduce multiple functions to the catalytic surface of ACF by catalytic growth of CNF, which can improve the performance of ACFs in such applications as SOx and NOx. Robert *et al.,*[18] grew carbon nanostructures (CNFs and carbon nanotubes) on a carbonaceous carrier (activated carbon) for use in water purification. The AC/CNF composite was more effective at purifying water than conventional AC, especially for removing metal species.

In surface related applications of carbon materials, surface morphology, structure, chemistry, and the degree of effective sites on their surface is very important. Platelet and herringbone CNFs expose free edges of hexagons with few heteroatoms, differing from a negatively concave surface of carbonaceous carrier based on a micropore-developing structure. Such a surface of CNF appears to be advantageous to providing active sites of exposed hydrophobic hexagon edges for high activity [19].

The scope of the present work was to prepare a composite from activated carbon and carbon nanofibers (AC/CNF) to improve the performance of activated carbon in such applications as adsorption of VOCs, and to use the prepared composite in granular form in respirator cartridges for respiratory protection.

## Materials and methods

### Activated carbon preparation

Iran is the third largest producer of walnuts in the world, with 10% of global output [20]. In this study, walnut shell was used as the carbonaceous source material for preparation of the activated carbon. Potassium hydroxide was used as the chemical activating agent as it is one of the most effective compounds for the production of activated carbons [21,22].

Walnut shells with an initial particle size of 0.21-0.35 mm were then crushed, milled, and screen-sieved. The shells were next put to soak in a solution of potassium hydroxide, gently heated for 2 hours. They were subsequently dried in an oven at 150°C. The impregnated shell samples were then carbonized in a quartz tubular reactor under a nitrogen flow for 1 hour. After activation, the samples were cooled down under N_2_ flow and washed out twice with a 0.5 N hydrochloric acid and rinsed sequentially with cold distilled water for the removal of any residual chemical substances. The washed samples were dried at 110°C in an oven [23]. Samples with different weight ratios of activation agent (potassium hydroxide) to walnut shell, and with different activation temperatures, were prepared, and the sample with highest surface area, micropore volume and VOC adsorption capacity, was used as the support material for preparing the AC/CNF composite.

### Impregnation of catalyst into AC and growth of CNF over AC

AC previously dried at 65°C was allowed to soak up a water solution containing a prescribed amount of nickel. After evaporation of most of the water, the damp AC was dried in a vacuum oven at 120°C for 2 hours [12,18]. Hereafter in this study, AC impregnated with 30% w/w of Ni is referred to as AC.

CNFs were synthesized over AC in a quartz flow reactor (diameter 3 cm, length 100 cm) heated by a conventional horizontal tube furnace. A prescribed amount of AC was placed in an alumina boat at the center of the reactor tube in the furnace.

After the reduction of catalysts in a hydrogen atmosphere at 500°C for 2 h, methane was flowed over the alumina boat at a rate of 200 cm^3^/min at 550°C for 1 h, and was catalytically decomposed. Nano-sized carbon grew on the AC carrier material, by chemical vapor deposition (CVD). To purify the AC/CNF samples as grown by this process an acid treatment was adopted to remove the catalyst particles. The samples were mixed with hydrochloric acid (3 M) for 12 h and then with nitric acid (2 M) for a further 12 h, with the aid of ultrasonication.

Finally the samples were rinsed with cold distilled water for the removal of residual chemical substances. The washed samples were dried at 110°C in an oven for 12 h [23].

The synthesized composite was physically activated by passing a controlled amount of CO_2_ over the composite suspended in a reactor tube in the furnace. The sample was heated under N_2_ flow, with the designated reaction temperature increasing by 5°C per minute and then maintained at a temperature of 450°C for 5 hours.

### Characterization of activated carbon and AC/CNF composite

The samples were subsequently degassed at 150°C for 8 h. Pore size distribution and specific surface area of the produced adsorbents were determined by nitrogen adsorption isotherm at 77°K, using a Micromeritics ASAP 2010 analyzer accelerated surface area and the Brunauer, Emmett and Teller (BET) and Barrett, Joyner, and Halenda (BJH) methods.

The nanostructure and morphology of the nanocomposites were observed under both scanning electron microscope (SEM, Cambridge S-360 operated at 16 kV and 2.5A) and transmission electron microscope (Philips CM-100 operated at 100 kV).

### (VOCs) adsorption

1000 ppm of BTXs, as representative VOCs, was used to determine the adsorption capacity of the powdered form of the prepared adsorbents , as this is a typical maximum use concentration (MUC) of organic vapor respirator cartridges [24].

For the adsorption study, model air samples were generated by injection of 84 microlitres of equal BTX mixture (using a 100μL syringe) into a 5 l Tedlar-bag previously filled with hydrocarbon-free air under ambient pressure. The injection valve was immediately closed and the bag was heated in an oven at 80°C for 1 hour. The adsorption studies were conducted using the following method [25].

Prior to the experiment, the samples were degassed at 200°C for 8 h to remove adsorbed moisture or gases trapped in the samples. The adsorbents were then homogenously packed into a 9 cm length of glass column (internal diameter 10 mm). Silane-treated glass wool and propylene filters were used at both ends of the column to hold the adsorbents in place. The final mass of adsorbent in each column was approximately 80 mg. The inlet end of the adsorbent-column was connected to the sample bag. The other end was connected to the charcoal sorbent tube (Sorbent Tube, Anasorb CSC, SKC) for trapping any adsorbate not retained by the adsorbents in the column.

The outlet end of the sorbent tube was connected to a SKC low flow pump. The air samples were passed through the whole system at an adjustable flow rate.

To measure the amounts of BTXs trapped by the charcoal, they were extracted using carbon disulfide (CS_2_). Analyses were performed using a Shimadzu Gas chromatography system. The BTXs adsorbed on the adsorbents were estimated by subtraction of the BTXs retained on the charcoal from the initial quantities injected into the sample bag. Each adsorption experiment was conducted in triplicate for each sample.

### Granular adsorbents for respirator cartridges

Because of difficulties in handling and breathing resistance, the powdered form of the prepared adsorbent was not appropriate to applications such as respirator cartridges. In order to convert it into a granular form, the adsorbent was mixed with a suitable carbonaceous binder (just enough to allow kneading and homogeneous mixing) and manually extruded. The granular adsorbent was then carbonized in an oven at 800°C overnight, and was finally activated with CO_2_ for 8 hours.

40 grams of prepared granular adsorbent with an average granule size of 0.11 cm and packing density of 0.43 g/cm^3^ was used for preparation of respirator cartridges with the bed depth and diameter of 3 and 7.4 cm respectively.

### Cartridge breakthrough time testing

**Figure 1:**
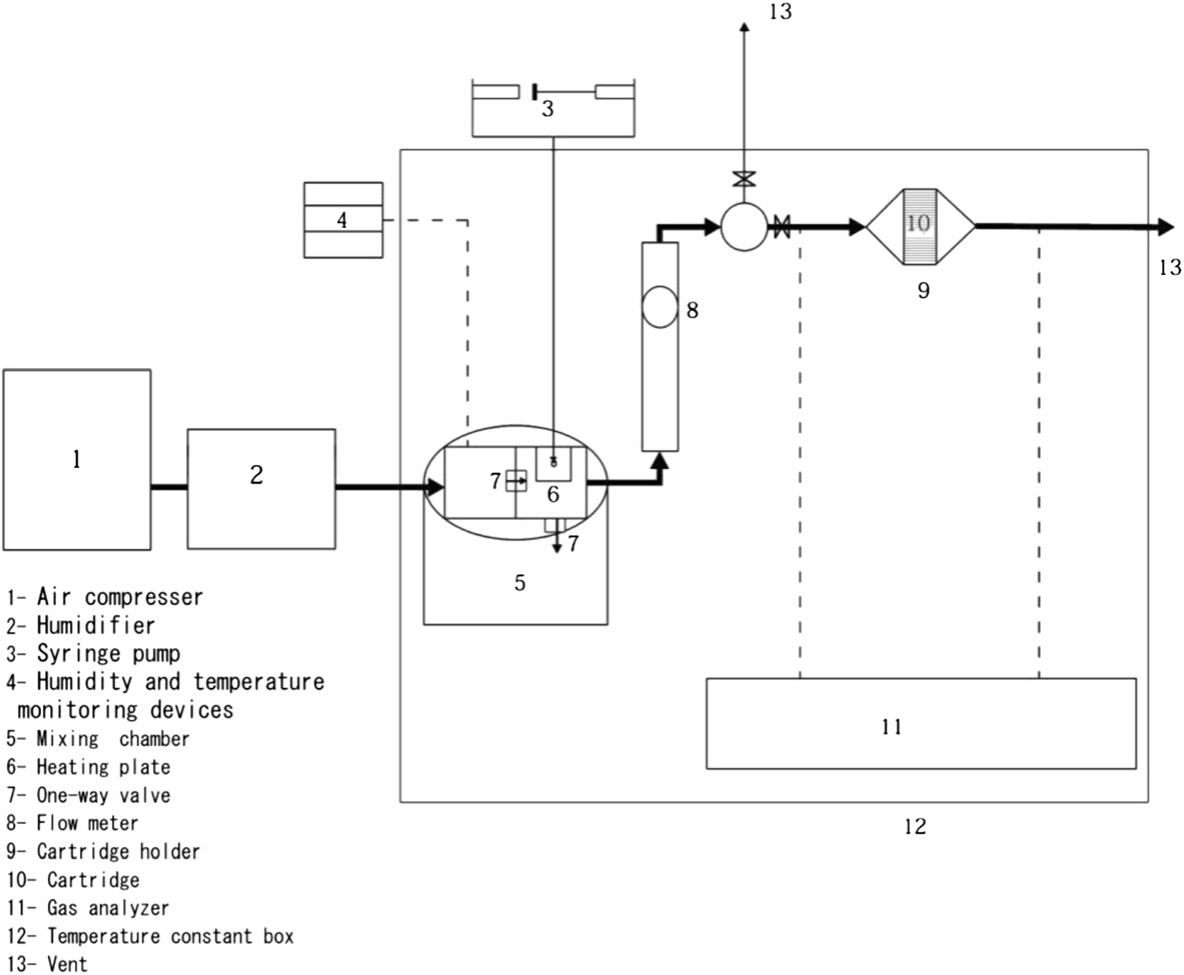
A schematic diagram of the apparatus used for measuring cartridge breakthrough time.

For measuring the breakthrough time of prepared respirator cartridges, they were exposed to cyclohexane (as a representative organic vapor) according to the EN 14387:2004 standard [26], with the aid of the apparatus illustrated in Figure 1.

The main part of this apparatus was the mixing chamber, which was made of PTFE and consisted of three parts. The first part was equipped with temperature and relative humidity sensors (TC4Y-14R, Autonics, Korea); the second part accommodated a heating plate (item 6) for vaporizing the solvent as it entered from the syringe pump (model HX-901A, item 3). After humidification, the air (item 2) passed through the first part to the second, mixed with vaporized solvent, and then entered the third part (item 5, the main part of mixing chamber).

These parts were clamped together with an o-ring to provide a leak-proof fit, and two one-way valves (items 7) between the parts prevented back pressure inside the chamber.

Inlet air was provided by an air compressor, filtered for organics and particulates, and passed through a humidifier to control and maintain a constant humidity in the mixing chamber. The air temperature and relative humidity were adjusted at 25±2.5°C and 70±2.5%, respectively.

The mixing chamber output was directed into the cartridge holder where the test piece was fixed. At the start of run the mixing chamber output was bypassed to a vent (item 13). After adjusting both the temperature and humidity, and after obtaining a steady state concentration based on the desired values (which normally took 30 min.), the bypass valve was shut, the air stream was allowed into the main line, and the test started.

The concentration of cyclohexane vapor was regulated in the range of 1000±10 ppm by controlling the injection rate of the syringe pump.

The vapor concentration of cyclohexane was measured both upstream and downstream of the cartridge, using a gas analyzer equipped with a Photo Ionization Detector (Ion Sciences Co., UK).

The interval between the start of the test flow and the time when the vapor concentration downstream of the respirator cartridge reached a value of 10±2 ppm was recorded as breakthrough time.

## Results

### Characterization and microscopic analyses of prepared adsorbents

**Figure 2:**
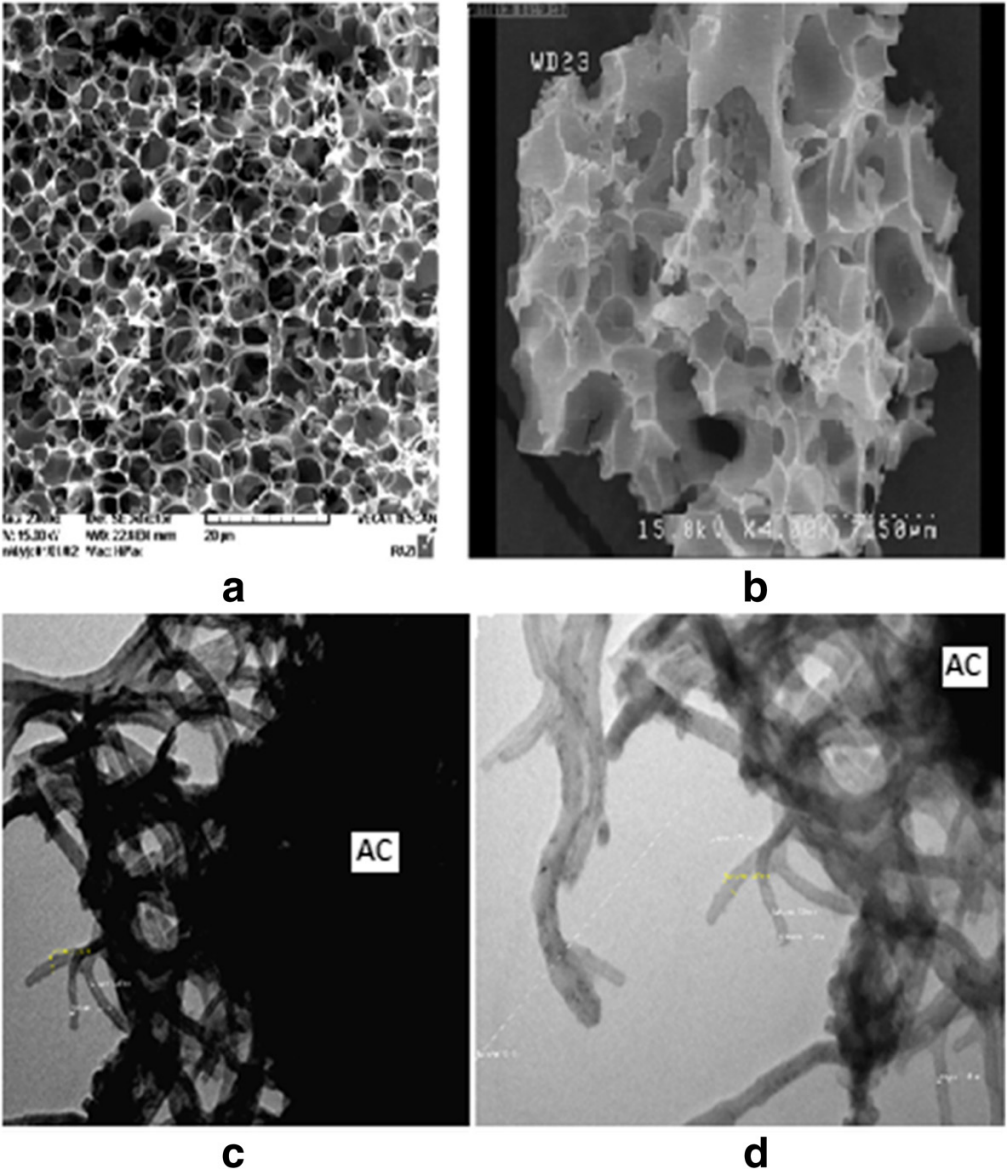
SEM photographs of prepared walnut shell activated carbon (a and b) and, TEM photographs of synthesized CO_2_ activated AC/CNF composite(c and d).

Figures 2a and 2b show SEM photographs of the porous structure in the optimum sample of prepared activated carbon, which was obtained through activation of walnut shell with potassium hydroxide at a weight ratio of 1.7 and temperature of 700°C.

Figure 2 (c and d) shows TEM photographs of the prepared AC/CNF composite. As it can be seen, CNFs with a very thin diameter of about 10-20 nm were formed uniformly on the AC/CNF. The diameter of CNF has been known to depend on the catalyst size [12,27,28].

### Surface area and pore distribution of AC/CNF composites

**Figure 3:**
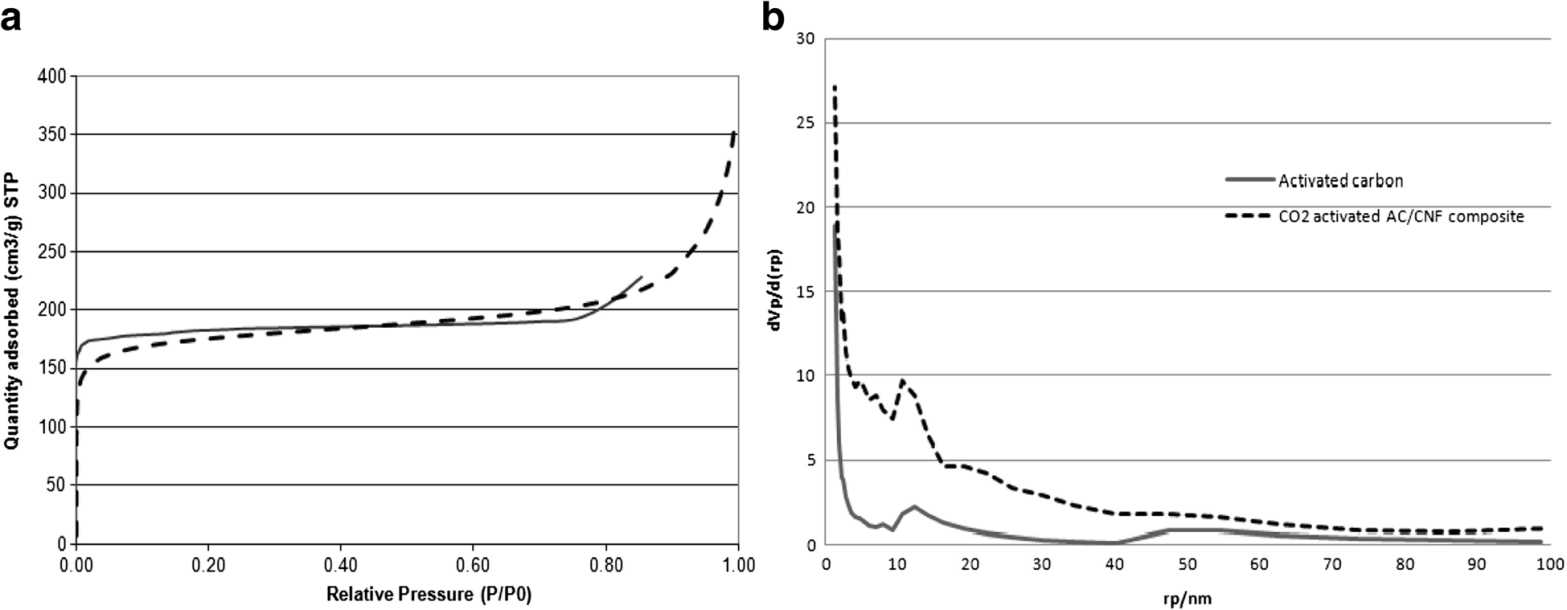
Nitrogen adsorption isotherms (a) and BJH pore size distribution of prepared adsorbents from AC and CO_2_ activated AC/CNF composite (b).

The nitrogen adsorption isotherms for the prepared samples are shown in Figure 3(a). Figure 3b shows the pore size distribution in the original AC and in CNF-grown ACs obtained by BJH analysis.There are more micropores with radii of less than 1 nm in the CO_2_ activated AC/CNF than in the original AC.

The BET specific surface area and the micropore volume of prepared adsorbents are summarized in Table 1. The optimum sample of prepared activated carbon had a surface area of 737 m^2^/g while after impregnation with the Ni catalyst and growth of carbon nanofibers on it, the surface area decreased to 422 m^2^/g with disappearance of the micropores.

**Table 1:** BET surface area and pore properties of prepared activated carbon and AC/CNF composites

**Sample**	**BET surface area (m^2^/g )**	**Pore volume (cm^3^/g)**	**Average pore diameter (Å)**	**Micropore Volume (cm^3^/g)**
Activated carbon	737	0.404	2.2	0.271
AC/CNF composite	422	0.024	16.02	0.07
CO_2_ activated AC/CNF composite	686	0.529	3.84	0.242

### Adsorption studies and cartridge breakthrough testing

**Figure 4:**
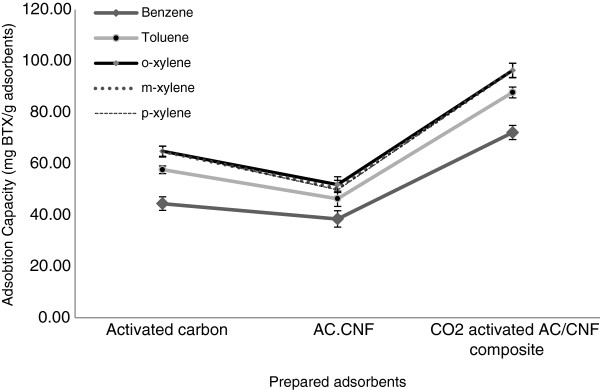
Absorption capacities of the prepared adsorbents.

The absorption capacities of the prepared adsorbents are illustrated in Figure 4. which show that the BTX adsorption capacity of the AC/CNF sample (before CO_2_ activation) was lower than the AC sample.

**Figure 5:**
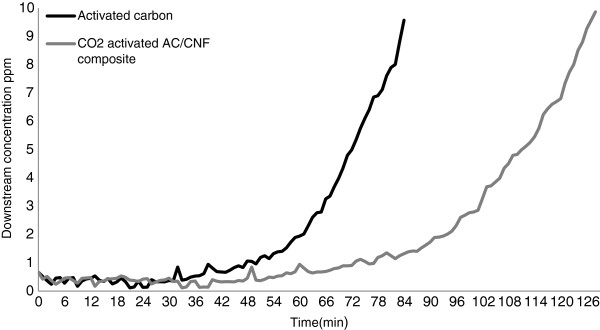
Cyclohexane breakthrough time of cartridges prepared from walnut shell activated carbon and CO_2_ activated AC/CNF composite.

Figure 5 show the outlet concentration of cyclohexane with cartridge exposure time. The cyclohexane breakthrough time with walnut shell activated carbon and CO_2_ activated AC/CNF were 85 and 117 minutes, respectively.

## Discussion

### Surface area and pore distribution of AC/CNF composites

As shown in Table 1 after impregnation of activated carbon with Ni catalyst and growth of carbon nanofibers on it, the specific surface area and the micropore volume have decreased. This indicates that the catalyst impregnated on the AC blocked most of the micropores, and the catalyst particles were selectively deposited on the pores of the AC. Thus, AC could not contribute significantly to the surface properties of AC/CNF composites in which AC appears only as a catalyst support or macroscopic frame. The reduction in the BET area after growth of CNF on activated carbon, has been reported in previous studies [12,29].

As observed from Table 1, AC mostly contains micropores, whereas after growth of CNF on the activated carbon (before CO activation) micropore volume has decreased significantly. Therefore, it may be inferred that the AC/CNF mainly possessed mesoporous and macroporous features.

After catalyst deposition and growth of CNF on AC, CO2 activation partially restored the surface area by regenerating the micropores with a smaller radii and a pore volume close to those of the original AC (Table 1). This activation resulted in a sharp increase of surface area with the development of slightly larger pores than those of the original AC and appeared to open most of the pores blocked by the catalyst particles, forming a different pore system [12]. Therefore, the physical activation step was found to be critical to express both surfaces of CNF and AC, and it seems that formation of CNF followed by CO_2_ activation created new pores with radii of smaller than 1 nm, although the catalyst particles still partially blocked the pores present on the surface of the AC.

Lim *et al.*[12] presented a schematic model of the overall step from catalyst deposition on AC, partial oxidation of AC containing the catalyst, and growth of CNF on the AC surface. The catalyst deposited on AC can be thought of as in three positions: catalyst particles in the pores, on or at the entrance of pores, or on the flat ‘external’ surface of AC. When the location of catalyst is at the pore entrance, or on the flat surface of the supporting material (AC), CNF yield will be much higher than when catalyst particles are in the pores of supporting material. In this position, it is difficult for the gas-phase carbon source to react with the catalyst and yield CNFs. In this study, with a reaction time of 1 h at 550°C, we were able to yield a composite adsorbent of 40% CNF (measured gravimetrically).

TEM of CNFs grown on AC (Figure 2) shows some entanglements and nodes along their axes, which lead CNFs to fill more open space in the AC pores. However, the high surface areas of 622 m^2^/g achieved by CO_2_ activation after CNF growth on the AC suggest that a large amount of micropores on AC still contribute the surface properties of the composites. Thus, free edges of CNF and micropores of AC are believed to jointly perform surface functions [12]. It seems that with the catalytic growth of CNFs on the activated carbon, a new surface and pore structures have been created on the both the porous surface of activated carbon and the exposed edges of CNF.

### Adsorption studies and cartridge breakthrough testing

The reduction in BTX adsorption capacity of AC/CNF sample (before CO_2_ activation) is related to the disappearance of micropores and a concurrent reduction in surface area. However, after CO_2_ activation, adsorption capacity has been increased remarkably, due to regeneration of micropores and creation of a different pore system, in comparison with the original activated carbon.

Usually, the carbons used in respiratory protective devices are originated from coconut shell or are petroleum based, because they exhibit highly developed microporous surfaces required for maximum adsorption [30]. In this work, respirator cartridges were prepared from walnut shell activated carbon and as shown in Figure 5, this substance alone could meet the minimum breakthrough time of 70 min for respirator cartridges as defined by EN 14387:2004 Standard. However, cartridges prepared from CO_2_ activated AC/CNF lasted about 50% longer than those cartridges prepared from walnut shell-based activated carbon alone with a same weight of adsorbents.

With the same bed diameter, an AC/CNF cartridge with a 2 cm bed depth exhibited the same breakthrough time as an AC respirator cartridge with a 3 cm bed depth. So, one of the advantages of these cartridges could be their lower weight in comparison to activated carbon cartridges.

## Competing interests

The authors declare that they have no competing interests.

## Authors’ contributions

The overall implementation of this study including design, experiments and data analysis, and manuscript preparation were the results of efforts by first three authors. The fourth and fifth authors has helped in synthesize and optimization of nanocompisites. Other authors have helped in experimental design and data analysis. All authors read and approved the final manuscript.
